# Efficacy, Effectiveness, and Quality of Resilience-Building Mobile Health Apps for Military, Veteran, and Public Safety Personnel Populations: Scoping Literature Review and App Evaluation

**DOI:** 10.2196/26453

**Published:** 2022-01-19

**Authors:** Melissa Voth, Shannon Chisholm, Hannah Sollid, Chelsea Jones, Lorraine Smith-MacDonald, Suzette Brémault-Phillips

**Affiliations:** 1 Heroes in Mind, Advocacy and Research Consortium Faculty of Rehabilitation Medicine University of Alberta Edmonton, AB Canada; 2 Department of Occupational Therapy Faculty of Rehabilitation University of Alberta Edmonton, AB Canada; 3 Leiden University Medical Centre Leiden University Leiden Netherlands; 4 Operational Stress Injury Clinic Alberta Health Services Edmonton, AB Canada

**Keywords:** occupational stress injury, trauma, mHealth, resilience, mental health, military, veteran, public safety personnel, OSI, PTSD, mental health intervention, mobile phone

## Abstract

**Background:**

Military members (MMs) and public safety personnel (PSP) are vulnerable to occupational stress injuries because of their job demands. When MMs and PSP transition out of these professions, they may continue to experience mental health challenges. The development and implementation of resilience-building mobile health (mHealth) apps as an emergent mental health intervention platform has allowed for targeted, cost-effective, and easily accessible treatment when in-person therapy may be limited or unavailable. However, current mHealth app development is not regulated, and often lacks both clear evidence-based research and the input of health care professionals.

**Objective:**

This study aims to evaluate the evidence-based quality, efficacy, and effectiveness of resilience-building mobile apps targeted toward the MMs, PSP, and veteran populations via a scoping literature review of the current evidence base regarding resilience apps for these populations and an evaluation of free resilience apps designed for use among these populations.

**Methods:**

The studies were selected using a comprehensive search of MEDLINE, CINAHL Plus, PsycINFO, SocINDEX, Academic Search Complete, Embase, and Google and were guided by PRISMA-ScR (Preferred Reporting Items for Systematic Reviews and Meta-Analyses extension for Scoping Reviews). A narrative synthesis of the resulting papers was performed. The Alberta Rating Index for Apps was used to conduct a review of each of the identified apps. The inclusion criteria consisted of apps that were free to download in either the Google Play Store or the Apple App Store; updated within the last 3 years; available in English and in Canada; and intended for use by MMs, veterans, and PSP.

**Results:**

In total, 22 apps met the inclusion criteria for evaluation. The resilience strategies offered by most apps included psychoeducation, mindfulness, cognitive behavioral therapy, and acceptance and commitment therapy. Overall, 50% (11/22) of apps had been tested in randomized controlled trials, 7 (32%) apps had been evaluated using other research methods, and 5 (23%) apps had not been studied. Using the Alberta Rating Index for Apps, the app scores ranged from 37 to 56 out of 72, with higher rated apps demonstrating increased usability and security features.

**Conclusions:**

The mHealth apps reviewed are well-suited to providing resilience strategies for MMs, PSP, and veterans. They offer easy accessibility to evidence-based tools while working to encourage the use of emotional and professional support with safety in mind. Although not intended to function as a substitute for professional services, research has demonstrated that mHealth apps have the potential to foster a significant reduction in symptom severity for posttraumatic stress disorder, depression, anxiety, and other mental health conditions. In clinical practice, apps can be used to supplement treatment and provide clients with population-specific confidential tools to increase engagement in the treatment process.

## Introduction

### Background

Globally, military members (MMs) and public safety personnel (PSP), for example, correctional workers, dispatchers, firefighters, paramedics, and police officers, experience increased exposure to trauma and stress in their daily activities, which can affect their mental health and well-being [1,2]. PSP and MMs are at an increased risk of developing occupational stress injuries (OSIs), including posttraumatic stress disorder (PTSD), major depressive disorder, generalized anxiety disorder, and increased anger, aggression, or hostility, which can lead to other challenges, such as substance abuse, relationship difficulties, and workplace absenteeism [1].

MMs in the Canadian Armed Forces (CAF) are at greater risk of mental health disorders and suicide risk compared with the Canadian civilian population [3]. Of the regular force CAF members, 32.2% self-reported a mental health problem related to emotions, stress, substances, or family in 2013-2014 [4]. In addition, within the 3-year period from 2013 to 2016, mental health conditions in the veteran populations showed an increase from 25.4% to 30.3%, with PTSD being the most commonly identified OSI [5]. A 2018 study indicated that across the Canadian PSP groups, 44% screened positive for at least one mental health disorder [1]. This study also found that 36.7% of surveyed Canadian police officers in particular screened positive for mental health conditions, primarily PTSD [1]. These populations face challenges related to attaining professional mental health, including displacement owing to relocation, working in remote geographic locations, and shift work.

Owing to the need for mental health support among MMs, veterans, and PSP, mobile health (mHealth) apps have emerged as a portable treatment modality option [6-8]. Interest and use of mHealth by clinicians has increased in recent years in health care practice [9]. The latest estimates suggest that there are between 165,000 and 325,000 health and wellness apps currently available for download [10,11]. When considering the MMs, veteran, and PSP populations, mHealth apps have gained popularity as a mental health treatment modality because of their low costs, easy access, and in-the-moment interventions [12].

### Resilience

Evidence illustrates that resilience training and interventions, primarily those focused on coping skills and self-efficacy, can work to support a decrease in psychological distress and symptoms of PTSD [13-16]. Resilience is a broad and often complex concept, which scholars have uniquely interpreted depending on the context and can encompass both the individual and the group [17]. For this study, we have defined resilience as follows: “The dynamic process of overcoming adverse experiences through the use of internal and external resources in order to foster healthy psychological functioning” [13,17-19].

**Figure 1:**
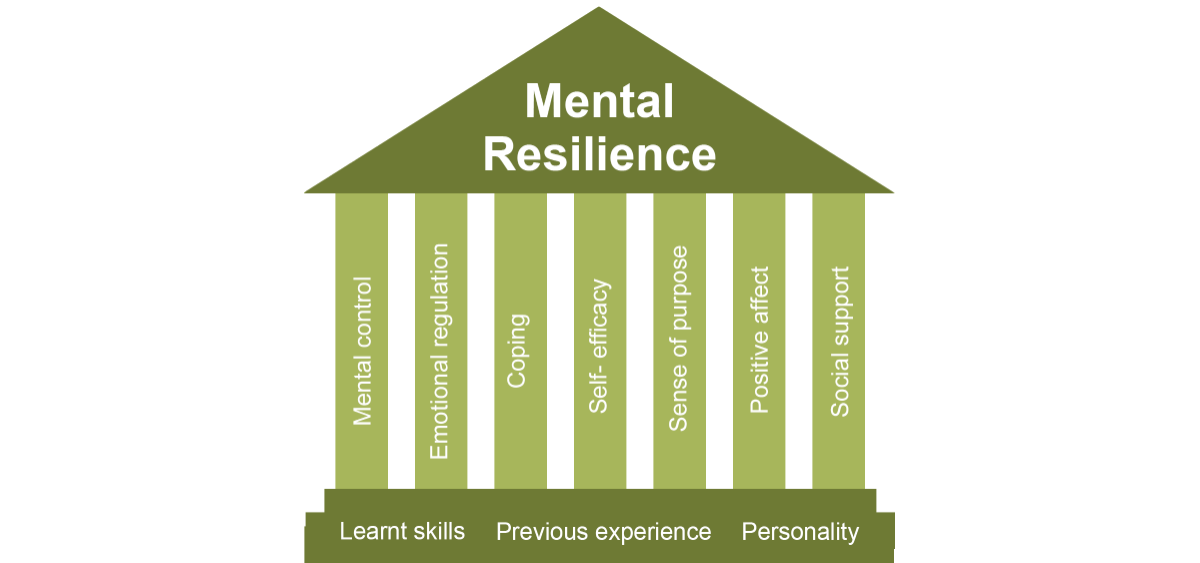
Pillars of mental resilience [21,22].

Resilience has been identified as an important factor that enables individuals adapt to and recover from emotionally, physically, and psychologically distressing situations and trauma [17,20]. There have been a multitude of resilience models and frameworks proposed in recent years specific to military, veteran, and PSP populations. One such model was developed using a large meta-analysis study by the Defence Human Capability and Science Technology Centre in 2014 [21]. This was further refined by Precious and Lindsay [22] with the Australian Armed Forces, resulting in a pillar of the mental resilience model (Figure 1 [21,22]). This model collaboratively draws on the best evidence related to mental resilience, highlighting both aspects outside one’s locus of control (ie, learned skills, previous experience, and personality) and the activities and skills within one’s locus of control (ie, mental control, emotional regulation, coping, self-efficacy, sense of purpose, positive affect, and social support) [22].

The activities and skills listed in this model have been attributed to the fostering of resilience, including those used to improve emotional regulation and coping, such as mindfulness, grounding, and self-talk; positive affect, such as purposeful leisure activities; and interpersonal relationships [23,24]. According to Lopez [23], “resilient individuals have a greater likelihood of engaging in healthy and productive activities and having a better quality of life.” If an individual does not possess the self-regulatory abilities and tools necessary to deal with distressing situations, it can impact all their areas of life, including sleep, quality of life, work, motivation, interest, and engagement in daily activities [24]. Resilience is, therefore, vital for the MMs, veteran, and PSP populations, as it supports continued engagement in both purposeful activities and increases the ability to adapt to the challenges of daily living.

### Digital Health

The term *digital health* refers to the use of electronic communication, services, and processes to deliver and facilitate health care services [25]. mHealth is a more recent subsegment of digital health that uses mobile technology to enable remote care and clinical health data collection. Digital health–based treatments have become a growing field of research and development for the MMs, PSP, and veteran populations. Popular modalities include virtual reality, web-based programs and games, and mHealth apps [26-28]. Current research indicates that these platforms are effective at reducing the symptoms of PTSD and other mental health disorders caused by exposure to trauma [28-32].

Specific to the MMs, PSP, and veteran populations, health care professionals (HCPs) are well-suited to use mHealth tools, such as apps, to supplement treatment and provide clients with immediate tools to help them overcome psychological impairment related to their traumas [9,31]. For example, if a military member or PSP encounters a stressful situation, the accessibility of apps can help them navigate their feelings in real time. In addition, mHealth apps may fill a gap for those who require mental health treatment but are faced with barriers, such as stigma to visible help-seeking, long waiting times, a high mobility of their jobs, and geographic restrictions [8,30,33,34], all of which have been noted as problematic for the MMs, PSP, and veteran populations. Many apps are also cost-effective and may be beneficial where therapy services are limited or unavailable [35,36].

Although mHealth tools have significant potential, several barriers limit their full uptake in the health care system. For example, some forms of technology, though widely used, may still not be available to everyone under all circumstances, for example, locations with unreliable or reduced cellular service, limited Wi-Fi access, and financial barriers may impede their use [37]*.* More importantly, there is a paucity of peer-reviewed research published regarding mHealth apps to determine their uptake, impact, or the best practices for their development and regulation [12,30,37]. When considering the use and development of mHealth apps, the limited number of research studies and agencies available to regulate this field impacts the ability to meet the current needs of this rapidly expanding industry [12,37]. As a result, a significant proportion of apps currently available for download have limited evidence of the effectiveness, efficacy, and safety of their use. This can make it difficult for HCPs to identify the best mHealth resources to recommend to MMs, PSP, and veterans in support of their care.

### Objectives

The aim of this study is to evaluate the evidence-based quality, efficacy, and effectiveness of resilience-building mobile apps targeted toward the MMs, PSP, and veteran populations. This is addressed through two objectives: (1) completion of a scoping literature review of the current evidence base regarding mental health apps for these populations and (2) evaluation of common free mental health apps designed for use among these populations. We then compare and triangulate the data from these 2 approaches. The determination of these factors will aid in improving the evidence base for mHealth apps to highlight their potential use for HCPs who may be providing mental health services.

## Methods

### Objective 1

#### A Scoping Literature Review

A scoping literature review was completed to explore the available literature on mHealth apps and their cultivation of resilience in MMs, PSP, and veteran populations. The PRISMA-ScR (Preferred Reporting Items for Systematic Reviews and Meta-Analyses extension for Scoping Reviews) was used to guide this scoping review and app search, both of which were conducted between December 18, 2020, and December 20, 2020 [38]. We selected the following electronic databases for the search: MEDLINE (Ovid interface), CINAHL Plus with Full Text (EBSCOhost interface), PsycINFO (Ovid interface), SocINDEX with Full Text (EBSCOhost interface), Academic Search Complete (EBSCOhost interface), and Embase (Ovid interface). We also used Google as a search tool to investigate gray literature in case it was not detected in the chosen database. Additional resources were manually selected to ensure a comprehensive retrieval of relevant studies that may have fallen outside of the predetermined search terms. The following steps were adhered to: (1) determination of the population, intervention, comparison, and outcomes research question, (2) determination of an eligibility criteria, (3) definition of search terms (Multimedia Appendix 1), (4) title and abstract screening, (5) full-text reading, (6) charting of the data, and (7) narrative synthesis. The final literature search took place on December 20, 2020.

#### Determination of Research Question

This literature review aimed to answer the following research question: What is the efficacy, effectiveness, and quality of mHealth apps on increasing resilience and self-regulatory strategies among MMs, PSP, and veterans?

#### Determination of Eligibility Criteria

The literature included in the search encompassed studies published from the year 2000 onward to account for the development of technology during this time. The articles had to address resilience or self-regulatory strategies. In addition, the articles were required to pertain to military, veteran, or PSP populations (Textbox 1).

**Eligibility criteria for scoping literature review.**

**Inclusion criteria**

- The search was limited to studies published from the year 2000 onward to include more current technology.
- Included articles focused on participants aged ≥16 years.
- Articles addressing resiliency, hardiness, or coping.

**Exclusion criteria**

- Data not pertaining to military populations or public service personnel.
- Studies published in languages other than English.
- No outcome of interest.

#### Definition of Search Terms

Keywords for the search were determined using three main concepts: specific population, resilience, and games (refer to Multimedia Appendix 1 for a full description of the search terms).

#### Title and Abstract Screening

After the removal of duplicate articles from the search results, a minimum of 2 researchers screened each article based on their titles and abstracts to determine further eligibility for the literature review. Articles that did not meet the eligibility criteria were excluded. Conflicts were discussed and resolved via team consensus.

#### Full-Text Reads

The screened articles were then read in full by a minimum of 2 researchers. Conflicts were discussed and resolved via team meetings and final eliminations were made. The remaining articles were included in the scoping review.

#### Charting of the Data

The type of evidence, population, funding, interventions, outcomes, and recommendations were extracted from each remaining article and recorded on a spreadsheet.

#### Narrative Synthesis

A narrative synthesis was performed by 3 researchers to summarize the findings of the different studies and evaluation results. Narrative synthesis refers to an approach that relies primarily on the use of words and text to summarize and explain the findings of multiple studies associated with reviews [39]. Narrative synthesis can be particularly helpful when studies are heterogeneous and organizing the data in a more numerical or statistical format would be inappropriate. To conduct the narrative synthesis, 3 researchers first reviewed the included study results and deductively organized them using the pillars of mental resilience as a guide. Additional information related to study methods, key constructs, and study outcomes was then synthesized together to form a coherent understanding of each topic. Finally, the researchers provided a summary of the included articles and their relevance to app evaluations.

### Objective 2: Evaluation of Available Mental Health Apps

The identified apps were chosen through the following steps: (1) identification of the apps addressed in the literature review, (2) establishment of eligibility criteria, and (3) search of the eligible apps by name in the Apple App Store or Google Play Store. Apps were then evaluated for overall quality using the Alberta Rating Index for Apps (ARIA) [40].

#### Eligibility Criteria

Apps included in the study were available for download in the Apple App Store or Google Play Store, could be set up in English, and were accessible within the geographic region of Canada. Apps chosen were intended for use primarily with MMs, PSP, and veterans; however, app use and availability could also extend to civilian populations. We included only free apps because evidence indicates that although 93% of smartphone users are likely to download an app, only 35.8% would be inclined to pay for an app [41]. Refer to Textbox 2 for the detailed eligibility criteria.

**Eligibility criteria for mobile health apps included in the study.**

**Inclusion criteria**

- Apps that were available on the Apple App Store and Google Play Store.
- Apps that were free to download.
- Apps that were intended for use by military members or public safety personnel.

**Exclusion criteria**

- Apps that were not free to download.
- Apps that were not available in the English language.
- Apps that were not available in Canada.
- Apps that were not yet released for public use or access.

#### Outcome Measure: ARIA

The ARIA (Multimedia Appendix 2 [40]) was used as a measuring tool to rate each app included in the study [40]. There are two versions of the ARIA: one for care providers and one for end users. Although the ARIA has yet to be vigorously studied, its uptake by health care systems at the regional and national level has been swift, likely owing to its ease of use, applicability, and both clinical utility and accessibility to the client population. The ARIA is available in both English and French, allowing it to be used across Canada, particularly with all PSP and members of the CAF and Veterans Affairs Canada (VAC). The tool has 2 sections, A and B, with multiple feature items to be rated between 0 and 4: 0 being *strongly disagree* and 4 being *strongly agree*. Section A was completed before downloading the app. Items in section A included purpose, trustworthiness, privacy, and affordability [42]. Once completed, scores were added up for section A to obtain a total out of 24 [42]. Section B is scored once the user has spent a minimum of 10 minutes using the app [42]. The items in section B include security, trustworthiness, ease of use, functionality, target users, usefulness, and satisfaction [42]. When completed, the rater added up the section to obtain a score out of 48. Sections A and B were then added together to determine an overall score out of 72, with higher scores indicating better performance and user experience. The care-provider version also features a 0-4 rating scale on whether the app would be recommended to possible users. This measure does not impact the overall score of the app [42].

The care-provider version of the ARIA was chosen to review the selected apps because of its ability to be implemented by users, caregivers, and HCPs. Each identified app that met the eligibility criteria was evaluated by 2 reviewers using the ARIA. Conflicts were resolved through discussion with all researchers to determine the final ARIA score.

## Results

### Objective 1: Scoping Literature Review

#### Study Selection

**Figure 2:**
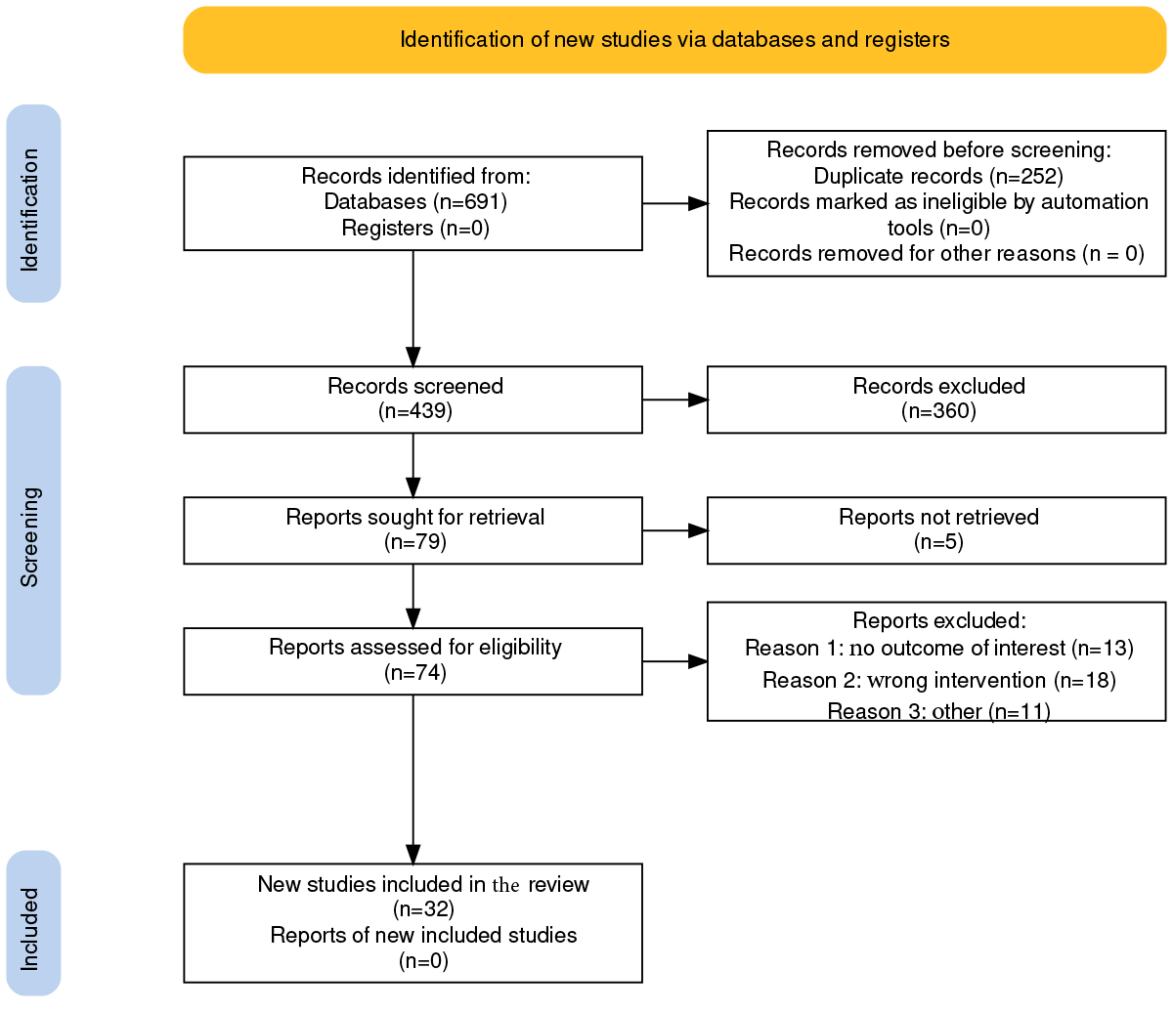
A PRISMA (Preferred Reporting Items for Systematic Reviews and Meta-Analyses) chart for the systematic review study identification, selection, exclusion, and inclusion.

After searching the databases and identifying records through additional sources, a total of 691 articles were identified. Seven additional records were identified from the other sources. After the removal of 252 duplicates, 63.5% (439/691) of articles remained for title and abstract screening. A total of 52.1% (360/691) of articles were determined to be irrelevant. Full-text reads were completed on the remaining 10.7% (74/691) of articles and 6.1% (42/691) of additional articles were excluded because of differences in outcomes of interest, irrelevancy to mHealth app use, limited qualitative or quantitative data, and repetitive publications. A total of 9 studies were excluded because they did not have a relevant outcome of interest. The reasons for exclusion were that the studies involved virtual reality without an app and were specifically for outcomes related to PTSD. From these 32 articles, 22 apps were identified as meeting the inclusion criteria. Figure 2 shows a PRISMA (Preferred Reporting Items for Systematic Reviews and Meta-Analyses) summary chart of the study selection process. The results of the scoping review, including narrative synthesis, are described in subsequent sections.

#### Study Findings

##### Evidence-Based Merit

Of the 22 apps identified in the scoping literature review, 11 (50%) apps had been tested in randomized controlled trials (RCTs), 7 (32%) apps using other methods, and 5 (23%) apps had not been research trialed at the time of this study. Alternative methods used to evaluate apps included a nonrandomized quasi-experimental pre–post follow-up design, pre- and posttest questionnaires, qualitative focus groups, and the Mobile App Rating Scale. Apps that were not research trialed were identified through scoping reviews. Two apps had been tested using multiple methods.

The literature indicated that out of the 22 apps, 15 (68%) used evidence-based strategies or incorporated evidence-based components within them. Among these 15 apps, 11 (73%) apps were developed using evidence-based practices as their foundation (*Virtual Hope Box, eQuoo, Mindfulness Coach, Mindarma, PE Coach, R2MR, High Res, PTSD Coach, CBT-I Coach, PHIT for Duty,* and *Stay Quit Coach*). In the RCT by Roy et al [28], 7 apps were included in determining the effectiveness of symptom reduction (*Positive Activity Jackpot, Tactical Breather, Daily Yoga, Simply Yoga, Life Armor, PE Coach,* and *Virtual Hope Box*). However, it should be noted that apps were not separated but rather assessed as a group, rendering it difficult to identify the efficacy and effectiveness of each app individually. *Virtual Hope Box and eQuoo,* were evaluated separately in their respective RCT studies. It was noted that *Mindarma* was utilized as a part of a mindfulness program for first responders. [15]

##### Mental Control, Emotional Regulation, Coping, and Self-efficacy

The resilience strategies offered by most of the apps fit within the pillars of mental resilience, including mindfulness training, psychoeducation, cognitive behavioral therapy, and acceptance and commitment therapy. Other strategies included biofeedback, sleep strategies, social engagement, mood tracking, time scheduling, and muscle relaxation techniques, such as yoga. Many of the apps included more than one strategy. Of the total 22 apps, 8 (36%) apps used mindfulness strategies, including *Mindarma*, *Mindfulness Coach*, *Daily Yoga*, *Simply Yoga*, *Virtual Hope Box*, *Tactical Breather*, *Breathe2Relax*, and *PHIT for Duty* and 4 (18%) apps encompassed psychoeducation: *eQuoo*, *Mindarma*, *Life Armor*, and *PE Coach*. Apps that applied cognitive behavioral therapy techniques included *R2MR*, *High Res*, *PTSD Coach*, *Family Coach*, *CBT-I Coach*, *Stay Quit Coach*, and *eQuoo*.

##### Effect of Apps on Resilience

The evidence-based literature demonstrated that many of the apps increased resilience strategies for users as well as improved the overall aspects of mental health [28,30,43,44]. The *eQuoo* app was demonstrated to significantly improve the traits of resilience, personal growth, and positive relationships [40]. In addition, *R2MR*, which stands for road to mental readiness, was found to be effective in increasing resilience and help-seeking behaviors in the participants and in reducing mental health stigma for individuals and entire workplaces [45]. Although much of the literature noted the integration of social support as a positive influence on app use, resilience and coping, and health promotion, there was an observed lack of social connectedness components within the apps reviewed [14,23,36,46].

##### Health Care and Social Support

Some apps, such as *PTSD Coach,* were demonstrated to be more effective in managing and reducing PTSD symptoms when used with the support of an HCP as opposed to independent use [43,47]. Sessions that provide instruction around optimal app use patterns as well as the app’s purpose, can increase the user’s knowledge and therefore adherence to treatment programs that uses mHealth components [43,48,49]. Additional mentoring or coaching may also contribute to a greater elaboration on techniques introduced within apps and may enable the transfer of skills from the app experience [14]. Evidence further indicates that using mHealth apps with support as supplementary resources, rather than primary treatment, may enhance therapeutic outcomes and allow users more autonomy in their ability to track symptoms while sharing results with their providers [14,30,43]. When users complete treatment sessions, they have the ability to retain these tools for future use or reference to their care [30,50].

##### End User Preferences, Incentives, and Real-world Apps

Within the studies, there were important themes that arose through narrative synthesis around user preferences. One of those themes included apps that had a sense of progression, rhythm and routine, and elements of personal causation [43]. Having set challenges and a clear visualization of the progress helped increase app use, adherence to the intervention, and goal attainment, especially when users were able to establish their targeted goals beforehand [26,51]. Earning rewards increased the attractiveness of the app, as did receiving guidance and instant feedback on target behaviors [26]. Apps designed around games and narratives were often preferred as they encompassed many of these traits and were user-friendly and enjoyable [51]. However, many users also noted a preference for more practical application opportunities of target skills, so their learned behaviors could be transferred to real-world concerns [52].

### Objective 2: Evaluation of Mental Health Apps

**Figure 3:**
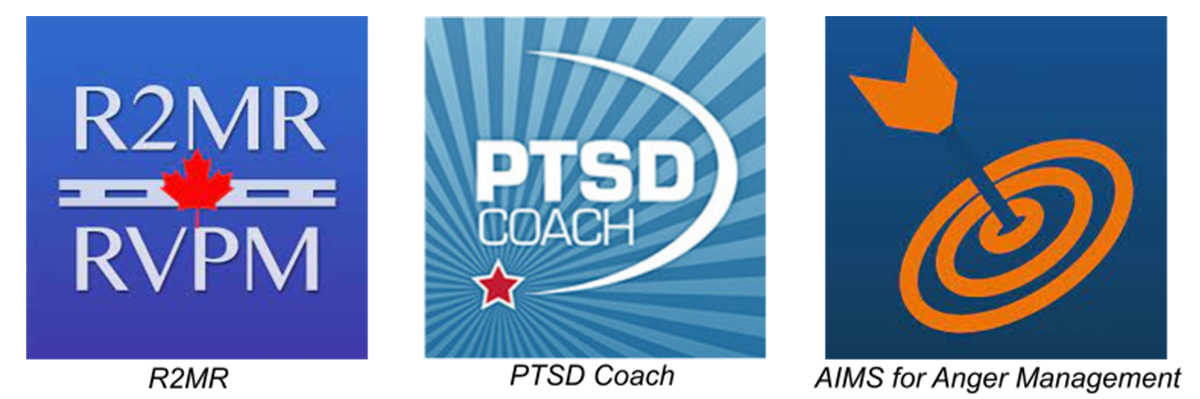
Highest scoring apps on Alberta Rating Index for Apps.

The ARIA scores ranged from 37 to 56 out of 72 (Multimedia Appendix 3). The highest overall scoring apps were *R2MR*, *PTSD Coach*, and *AIMS for Anger Management*, all of which had a total score of 56 (Figure 3). The lowest scoring apps were *Mindarma* (37 out of 72), *Breathe2Relax* (38 out of 72), and *High Res* (39 out of 72).

Certain items of the ARIA were identified as being particularly relevant to the MMs and PSP populations. These included security and confidentiality, trustworthiness, and usefulness and satisfaction. As security and confidentiality are a high priority for the MMs and PSP populations, the security item of the ARIA is imperative to acknowledge [30]. The following apps rated the highest on the security items with a 4 out of 4 rating on both security and consent: *Virtual Hope Box, Positive Activity Jackpot*, *Daily Yoga,* and *Life Armor*. The apps that were rated 1 or less were *eQuoo*, *Mindarma*, *High Res*, and *PHIT for Duty*. Many apps were missing features such as a password or biometric identifier and instead relied on the user’s phone security setup to provide these privacy measures.

Apps were rated higher on trustworthiness if developed by reliable sources with proof of evidence. In total, 15 apps were developed by government agencies in Canada and the United States, including the Department of Veteran Affairs, the Department of Defence, the National Centre for Telehealth and Technology, and VAC. For trustworthiness, *PTSD Family Coach, R2MR, Stay Quit Coach, CBT-i Coach*, and *eQuoo,* all scored 4 out of 4. Many apps did not state risk warnings associated with app use directly on their download page; however, information could occasionally be found within the app itself. For these cases, the apps were rated 1 out of 4 in trustworthiness. In the usefulness and satisfaction items, apps that rated the highest included *Mindfulness Coach* and *PTSD Coach*, both with combined scores of 11 out of 16 for the sections.

## Discussion

### Summary of Evidence

The aim of this study is to evaluate the evidence-based quality, efficacy, and effectiveness of resilience-building mobile apps targeted toward the MMs, PSP, and veteran populations. This involved the completion of a scoping literature review of the current evidence base regarding mental health apps for these populations and the evaluation of common free mental health apps designed for use among these populations. This study aims to provide some insight into the following research question: What is the efficacy, effectiveness, and quality of mHealth apps on increasing resilience and self-regulatory strategies among MMs, PSP, and veterans?

Overall, the results of this study indicated that most of the mHealth apps reviewed were well-suited to provide resilience strategies and skills for MMs, PSP, and veterans. These apps provided skills, strategies, and services, which could be categorized into the pillars of mental resilience and other commonly accepted definitions regarding psychoeducational interventions that can foster resilience. Common resilience strategies were well represented in many of the apps, often including mindfulness, psychoeducation, and positive coping or thinking skills. Our results indicated that no app fully addressed the 6 pillars of resilience identified by the armed forces of the United Kingdom and Australia. In total, 5 apps addressed 5 of the 6 pillars, whereas 12 apps addressed 4 or more of the 6 pillars, and 20 apps addressed 2 or more of the 6 pillars. The apps that rated the highest on the ARIA were *R2MR, Virtual Hope Box, eQuoo, Mindfulness Coach, and PTSD Family Coach.* The pillar most likely to be missing was *social* *support*, with the apps largely ignoring this concept.

In the evaluation of the 22 apps, *R2MR*, *PTSD Coach*, and *AIMS for Anger Management* had the highest overall scores. Points were generally lost because of a missing statement of the risk of use of the app or a lack of security measures to protect app access from the individual’s phone. Of the 22 apps assessed, 15 (68%) apps were developed by credible, military-focused government agencies (eg, VAC and the US Department of Veterans Affairs), which may help ensure that the content delivered was well-adapted for these populations. All but 2 of these apps were developed outside of Canada, which may impact the accessibility to local resources and services owing to the geographically based content (eg, helplines). Future comparisons of the ARIA with other app evaluation tools, such as the Mobile App Rating Scale, may allow for a more in-depth understanding of mHealth apps; however, for the MMs and PSP populations, the ARIA’s additional security and privacy questions provide a clearer understanding of population-specific concerns [7,44].

When considering evidence around the apps, it was noted that out of the 22 apps, only 11 (50%) apps had undergone evidence-based evaluation through an RCT. Although from the total 22 apps, only 11 (50%) apps were determined to be evidence-based, 15 (68%) apps had used evidence-based strategies or components within them. Some of the apps selected for evaluation in this study have not been evaluated in the evidence-based literature. This is partially a result of the large creation and turnover of mHealth apps available for download as well as the currently limited regulations guiding their development [7,12,30]. With this being an understudied area of research, a lack of evidence influenced other potentially important client considerations. For example, parameters around effective dosages of apps, such as how long and how often a user was required to use the tool to see lasting effects, were not addressed. Instead, many of the apps identified in the studies relied on user feedback to conclude whether the application was clinically effective [50]. Another usability component considered through the ARIA was the presentation of information. Many of the apps relied on large blocks of text to present educational information and, as noted by O’Toole and Brown [30], this format can be overwhelming for users and cause disadvantages for those with alternative learning styles. This review illustrates the limitations of both the evidence and the potential quality of the apps being proposed to support resilience in MMs, PSP, and veterans.

Although these apps incorporate strategies and skills that may assist in facilitating resilience, it must be acknowledged that there are other factors that impact their ability to increase foster resilience, such as individual motivation, education on use of the app, access to social support, and the use of apps together with an HCP or independently. In addition, care should be taken regarding the specific designation of these apps as *resilience apps* (particularly in light of the MMs, PSP, and veteran populations). As a universal operational definition of resilience is lacking, a clear understanding of what elements constitute or are most important to resilience is also lacking. To illustrate, the Canadian studies of resilience in MMs have independently and not always cohesively explored the constructs of personality, positive affect, mastery, and social support [53]; neuroticism, military hardiness, and problem-solving coping [54]; and conscientiousness, emotional stability, and positive social interactions [55]. This inability to effectively define what is meant by resilience within a specific organization, such as the CAF, is problematic for both the research and intervention development. This problem is compounded when exploring how the construct is defined by the militaries of other countries (ie, Australia, United Kingdom, and United States vs Canada) and how it may enhance mental health [56]. Without research to definitively measure resilience before and after app use and without an effective definition of resilience—which impairs the researcher’s ability to quantify the concept—it is not possible to decisively conclude whether these apps increase resilience.

### The Role of HCPs in mHealth and Resilience

Mental health can impact the daily functioning of an individual, and the concept of resilience is closely tied to mental health and well-being [23]. MMs and PSP are more likely to be exposed to traumatic experiences and are well-suited to resilience interventions [4,5]. HCPs can support individuals in navigating the environment for external resources and addressing the barriers in multiple domains that they may be experiencing.

Smartphones and technology are part of daily habits in the modern era, and HCPs can identify how to incorporate mHealth apps into health care settings. As MMs, PSP, and veterans face many unique challenges in terms of sudden environmental, lifestyle, and role changes, mHealth tools can present a more feasible option for access [24]. In a clinical context, HCPs can recommend apps to provide ongoing support outside of therapy services, create a tool for sharing health information, increase engagement in the treatment process, and sustain benefits gained once the provided services end [28]. For the mHealth apps themselves, the inclusion of both clinicians and users in their development can both ensure strategies meet the needs of their clients, thus encouraging wider acceptability and utility and helping clinicians better identify evidence-based features [30,57,58].

HCPs have a responsibility to advocate for best practices; this can be challenging with mHealth because of the high rate of app development and the inability for evidence-based practice to keep up [12,30,37]. Using tools such as the ARIA can help clinicians determine an app’s usability and evidence base; however, HCPs must also ensure that the app is a good fit for clients in terms of their interests, values, abilities, and routines. HCPs can then use this information to collaborate with the client, customize apps that meet the client’s needs and interests, and promote engagement with the apps. Creating client autonomy through app literacy will allow users to take more control in choosing treatment methods and encourage greater client-centered practice [59]. It is important to note, however, that many of these apps perform best when combined with the services of HCPs. This may include providing learning sessions before use to increase efficacy and optimal use patterns, weekly phone check-ins, or using the apps to enhance existing therapeutic interactions between clinicians and users [43,48-50]. In much of the literature, gamification and the integration of social components strengthened mHealth app use and engagement, resulting in more positive outcomes and an increased sense of peer support for the MMs and PSP populations [14,36,46]; however, this was lacking in practice.

### Strengths and Limitations

This study has several strengths. Both the scoping literature review and app evaluation were conducted following a planned a priori procedure, with attention to ensuring quality control and minimizing bias. The detailed search strategy in the literature review was extensive, including 6 databases. The inclusion and exclusion criteria were determined before the study onset and adhered to throughout for both the literature review and app evaluation. We also used appropriate calibration and at least two independent reviewers for all stages of the process.

There are certain limitations to this study, which should also be acknowledged. In the literature review, only studies written in English were included. The app selection criteria were limited to apps available for download in Canada, which excluded potentially beneficial apps available in other geographic locations. In addition, as the researchers only had access to iPhones, the apps were not tested on Android devices, which could have an impact on the usability criteria.

Although the authors identified the ARIA as an appropriate evaluation tool to use, it should be acknowledged that this is still relatively new and has not yet been extensively researched, used, or validated at this point. As such, there are currently no similar studies conducted with the ARIA, with which the present results could be compared. The results listed within this study only reflect app use from the perspective of clinicians, which could create bias. It will be important in future studies to invite users from the MMs, PSP, and veteran populations to complete the *user version* of the ARIA.

### Future Research and Directions

Regarding the future of mHealth and resilience, there is much work to do in the areas of research, development, and policy. First, despite its use in health care contexts, future research is required to determine how the ARIA scores correlate to app adherence, acceptance, and adoption by users as well as to health outcomes. Further comparison of the ARIA with other app evaluation tools would be valuable in understanding the utility, criterion validity, and other concepts related to its ability to rate apps from the perspective of HCPs and clients as end users.

As previously mentioned, there is a paucity of evidence-based studies on the existing apps geared toward both resilience and the specific patient population of MMs, PSP, and veterans. Although this lack of research does not necessarily indicate that apps are of poor quality, it highlights the need for further research on health app development to ensure safety, effectiveness, and efficacy. It has been identified that traditional study design and research methods may be inappropriate for the study of mHealth as technology evolves much faster than traditional evidence-based research [8,60]. The lack of empirical research to demonstrate the effectiveness of apps on resilience may be related to the short time frame during which mHealth apps have emerged and the speed at which their availability changes [8]. Innovative and novel research methods that can address the demands of mHealth and the rapidly changing world of app development and use are needed to assist with the quality control of these tools used by both HCPs and patient populations.

Another area of study related to mHealth that is important and specific to military and PSP users is privacy, security, and confidentiality. Although there is a higher expectation of privacy for apps that involve health care information, military and PSP organizations may be subject to other restrictions on internet access that may impede the use of the app or demand higher security. Data sharing and privacy are considerations that require attention from researchers, HCPs, and the general public when deciding on which app to use or if app use is appropriate at all [8]. Future systems of app evaluation and research would benefit from adding a component that considers data sharing, storage, and privacy in highly sensitive and secure patient populations (ie, military and PSP).

Future initiatives to assist HCPs and their clients in navigating the world of mHealth would be an asset to balance client autonomy through app literacy and would assure that apps have some level of evidence-based merit. As mHealth use is on the rise among many HCP and client populations, training to use apps to support service delivery is of utmost importance for health care organizations. The establishment of clear practice guidelines is important for both HCPs and clients, so that expectations about the usefulness and effectiveness of the app are appropriately managed. Currently, clients may have overly enthusiastic ideas about the effectiveness of these apps to develop their resilience and support their mental health, even when seeking professional mental health treatment might be necessary. Similarly, issues of risk and safety in mental health apps (including resilience) also need to be addressed.

The question of what constitutes a resilience app versus, for example, a wellness app, is also a murky territory that requires further navigation. Until a universally operationalized definition has been established for resilience, it is likely that confusion will remain regarding the codification of specific resilience skills and strategies. For example, family- and community-level factors were seldom addressed in our identified apps, despite being indicated as an important component of resilience [44,46]. This lack of inclusion is more surprising given the strong evidence that social support is a strong contributor to psychological health [23,24], overall well-being, and quality of life [24]. Similarly, empowering others to use their skills for stress reduction, coping, and building self-efficacy has been demonstrated to foster a significant reduction in the severity of the symptoms of PTSD, depression, and anxiety [12,13,28,58], which also alludes to the idea that the identified apps could be classified in terms of specific mental disorders. Until researchers, app developers, the various military and PSP organizations, and HCPs can agree on the definitions of these concepts, determining which intervention targets and affects resilience will remain elusive.

Finally, the evaluation of resilience apps will remain challenging for all stakeholders and can affect the quality of the product unless all stakeholders were included in the consultation process. With the exception of the apps designed by the United States Department of Defense of Veterans Affairs, it was difficult to determine if the end user’s perspective was incorporated into the development of the app. A collaborative approach to development, using both expert and user input, has been noted in recent studies as an effective approach to increasing the success of apps [57,58,60]. Ideally, in the future, mHealth development should engage the end users’ input to assist with contextualization, which may increase the app acceptance, usability, and feasibility in a multitude of health care and possibly military or PSP settings.

### Conclusions

Resilience is often targeted by HCPs through interventions that strengthen social support systems, foster greater self-concept, encourage optimism, promote the ability to reflect, and build emotional strength. Although not intended to function as a substitute for professional services and interventions, mHealth apps have the potential to foster resilience and support a significant reduction in symptom severity for OSIs, including PTSD, depression, and anxiety, in populations affected by OSIs, such as MMs, veterans, and PSP. Apps provide easy accessibility to evidence-based tools and encourage users to initiate help-seeking behaviors when stigma or uncertainty may impede the use of direct care. In clinical practice, HCPs can assist clients in identifying apps that support their habits and values and bolster participation and engagement in activities of daily living. As accessible, novel, and evidence-based interventions and resources for fostering resilience and addressing mental health become available, MMs, veterans, and PSP may be able to facilitate their healing, recovery, and growth, which would have a positive effect on their families, communities, organizations, and the public they serve.

## Appendix

Multimedia Appendix 1 Literature search strategy for scoping review.

Multimedia Appendix 2 Alberta Rating Index for Apps care-provider and user versions.

Multimedia Appendix 3 Alberta Rating Index for Apps score table.

## Abbreviations

ARIA: Alberta Rating Index for Apps
CAF: Canadian Armed Forces
HCP: health care professional
mHealth: mobile health
MM: military member
OSI: occupational stress injury
PRISMA: Preferred Reporting Items for Systematic Reviews and Meta-Analyses
PRISMA-ScR: Preferred Reporting Items for Systematic Reviews and Meta-Analyses extension for Scoping Reviews
PSP: public safety personnel
PTSD: posttraumatic stress disorder
RCT: randomized controlled trial
VAC: Veterans Affairs Canada
