# Correction: Efficacy, Effectiveness, and Quality of Resilience-Building Mobile Health Apps for Military, Veteran, and Public Safety Personnel Populations: Scoping Literature Review and App Evaluation

**DOI:** 10.2196/51609

**Published:** 2023-08-28

**Authors:** Melissa Voth, Shannon Chisholm, Hannah Sollid, Chelsea Jones, Lorraine Smith-MacDonald, Suzette Brémault-Phillips

**Affiliations:** 1 Heroes in Mind, Advocacy and Research Consortium Faculty of Rehabilitation Medicine University of Alberta Edmonton, AB Canada; 2 Department of Occupational Therapy Faculty of Rehabilitation University of Alberta Edmonton, AB Canada; 3 Leiden University Medical Centre Leiden University Leiden Netherlands; 4 Operational Stress Injury Clinic Alberta Health Services Edmonton, AB Canada

In “Efficacy, Effectiveness, and Quality of Resilience-Building Mobile Health Apps for Military, Veteran, and Public Safety Personnel Populations: Scoping Literature Review and App Evaluation ([JMIR Mhealth Uhealth 2022;10(1):e26453]) the authors made the following 4 corrections:

1. In the originally published article, in 8 instances the name of an app appeared as:

Resilience@Work/Mindarma

This has been corrected as follows in all 8 instances:

Mindarma

2. In the originally published article, in the Results: Study Findings: Evidence-Based Merit section, the last sentence appeared as follows:

Virtual Hope Box, eQuoo, and Resilience@Work/Mindarma were evaluated separately in their respective RCT studies.

This has been corrected as follows:

Virtual Hope Box and eQuoo, were evaluated separately in their respective RCT studies. It was noted that Mindarma was utilized as a part of a mindfulness program for first responders [15].

3. In the originally published article, in the Results: Study Findings: Mental Control, Emotional Regulation, Coping, and Self-efficacy section, the following sentence appeared:

Resilience@Work/Mindarma was the only app in this study that drew from acceptance and commitment therapy principles.

This sentence has been deleted from the paper.

4. In the originally published article, in the Results: Study Findings: Effect of Apps on Resilience section, the following sentences appeared:

Similarly, Resilience@Work/Mindarma showed improved adaptive resilience and psychological flexibility [15]. Joyce et al [15] also found that this app significantly increased optimism and mindfulness practice among study participants. This study also found that the app increased use of emotional support from others and help-seeking behavior, which addresses the social support pillar of the pillars of mental resilience.

These sentences have been deleted from the paper.

The correction will appear in the online version of the paper on the JMIR Publications website on August 28 2023, together with the publication of this correction notice. Because this was made after submission to PubMed, PubMed Central, and other full-text repositories, the corrected article has also been resubmitted to those repositories.
