# Supplementary material for: Efficacy, Effectiveness, and Quality of Resilience-Building Mobile Health Apps for Military, Veteran, and Public Safety Personnel Populations: Scoping Literature Review and App Evaluation
Source: JMIR Mhealth Uhealth. 2022 Jan 19;10(1):e26453. doi: 10.2196/26453 (PMC8811698; doi:10.2196/26453)
Supplement: Multimedia Appendix 1 [file mhealth_v10i1e26453_app1.docx]

# Multimedia Appendix A: Literature Search Strategy

The following databases were searched:

- MEDLINE (Ovid interface)
- CINAHL Plus with Full Text (EBSCOhost interface)
- PsycINFO (Ovid interface)
- SocINDEX with Full Text (EBSCOhost interface)
- Academic Search Complete (EBSCOhost interface)
- Embase (Ovid interface)
- Google (grey literature search)

Key search terms:

*Concept 1*:

- "Public Safety Personnel" or PSP or police* or firefighter* or firem*or military or soldier* or army or "special force*" or "active duty" or paramilitary or armed-force*or armed-service* or servicewoman or servicemen or air-personnel or defence-force* or defence-force* or service-personnel or navy or air-force or infantryman or Civil Defence or "medic" or active duty or enlisted personnel or reserve personnel or police* or RCMP or officer* or EMT or EMTs or EMS or paramedic*)

*Concept 2*:

- Resilien* or coping or hardiness or grit*

*Concept 3*:

- Game* or gaming or play or "group games" or "group activit*" or "augmented realit*" or "virtual realit*" or videogam* or VR or apps or app
