# Supplementary material for: Efficacy, Effectiveness, and Quality of Resilience-Building Mobile Health Apps for Military, Veteran, and Public Safety Personnel Populations: Scoping Literature Review and App Evaluation
Source: JMIR Mhealth Uhealth. 2022 Jan 19;10(1):e26453. doi: 10.2196/26453 (PMC8811698; doi:10.2196/26453)
Supplement: Multimedia Appendix 2 [file mhealth_v10i1e26453_app2.pdf]

Alberta Rating Index for Apps (ARIA)

User Manual

We are preparing a manuscript for publication soon. Meanwhile, please cite this work as follows:

Azad Khaneghah, P., Roduta Roberts, M., Stroulia, E., Ferguson-Pell, M., and Liu, L. (2020). Alberta Rating Index for Apps (ARIA) [measurement Instrument], Unpublished instrument.

Alberta Rating index for Apps, is a user-oriented index to help users of mobile health apps including patients, caregivers, and healthcare workers to evaluate the quality of mobile health applications. The index has two versions: The care-provider version and the user version.

The care provider version is designed for health care workers and family caregivers who can use this version to rate the quality of mobile health apps, as a proxy, on behalf of their patients or family members to who they provide care. When using this version, raters must consider the abilities and needs of the person who will ultimately use the app (e.g., a patient or a family member under your care).

The user version is designed for individuals who are interested in using mobile health apps for their own benefit. They can use this version to rate the quality of mobile health apps form their own point of view. Raters should consider their own needs and abilities when responding to the items.

Both versions have two sections: A and B. Raters must complete section A before they download the app. Try to answer all items in section A based on the information that you find on the app download page on the app store. Although section A is relatively short, do not rush through its items. It may take some time to find all the information you need to answer the items in this section.

After you answered all items in section A, add up the scores you gave to each item. It will range between 0 and 24, and the higher this score the better. If the results are satisfactory to you, you may proceed with downloading the app. Currently, we do not recommend any cut-off scores for section A. The decision to proceed with downloading an app depends on your subjective evaluation of the app. For example, if you do not find the purpose of the app in line with your objectives that is indicated by a score less than 3 on item 1 (i.e., the app is not useful to you) you may not even bother continuing with the rest of the evaluation. In another situation, you may find the app useful and from a trustworthy source, but at the same time you find the app to be very expensive (a score of 0 or 1 on item 6).

If you decide to download the app, then you can complete section B of the index. First read the items once to have a general idea about what to look for in the app. Next, use the app for as

long as you need to become familiar with all features of it. The instructions at the beginning of section B sates: “use the app for at least 10 minutes”. Please note that 10 minutes is the bare minimum time for each app. Raters are advised to work with the app for as long as they feel comfortable that they have discovered all features of the app. For some apps you may need to wait a couple of days to test if the reminders work properly. For some you may need to collect data for a few days to evaluate if the graphs work properly and are easy to understand. Do not rush to complete section B.

After you answered all the items in section B, then add up the scores you gave to each item. To calculate the total score of the index, sum up the scores for section A and section B. The higher the score, the better the app. Likewise, currently we do not recommend a cut-off for the total score of the index.

If you have any questions about the index, please email me at azadkhan@ualberta.ca.

I will appreciate your feedback and comments about ARIA.

Alberta Rating Index for Apps (ARIA)-Care provider version

App Name: ………………………………………….. I have used this app before: Yes ☐ No ☐

Use this index to rate the quality of a mobile health app for use by a client or patient (referred to as user). Please complete Part A and Part B.

Part A:

Complete this part before downloading the app on your phone or tablet.

- First, **find the app on your online app store**. For Apple products open "App store" for Android products go to the "Play Store."
- Then, read the app description from the online app store and rate the extent to which you agree with the following statements.

		Strongly disagree	Disagree	Neutral	Agree	Strongly agree
Purpose	1. The description of the app's purpose fits the user's goals. **(You may find app description under "About this app" on Google Play; or "Preview" on iTunes)**	0	1	2	3	4
Trustworthiness	2. Based on the description provided on the app store or the app developer's website, the user can trust that relevant experts in the field have developed the app.	0	1	2	3	4
	3. The app description includes a statement about the risks associated with using the app.	0	1	2	3	4
	4. The app declares conflicts of interest, if any.	0	1	2	3	4
Privacy	5. The app has a privacy policy that explains: (1) what information is collected by the app, (2) who will have access to this information, and (3) how this information will be used. **(Look for the "Privacy policy" of the app under the app description on "App Store" for Apple products or "Play store" for Android products)**	0	1	2	3	4
Affordability	6. The costs associated with using the app, including in-app purchases and subscription renewal fees, are affordable for the user. **(Go to the "App Store" for Apple products or "Play store" for Android products to learn how much does it cost to use the app)**	0	1	2	3	4
**Add up the scores for Part A**:……………………./24						

**Part B:** Use the app for at least 10 minutes or as long as you feel it is necessary to become familiar with its features. Try all **the links** and **buttons** on the screen. After you become familiar with the app, rate the extent to which you agree with the following statements. **You may go back to the app and check the features**.		Strongly disagree	Disagree	Neutral	Agree	Strongly agree
Security	1. The app uses at least one security measure, such as **user name and password** or biometric identifiers **(fingerprints, face recognition)**, to allow the user to access the app.	0	1	2	3	4
	2. The app asks for the user's consent if it needs to access the phone's camera, microphone, user's location, contacts, or photos.	0	1	2	3	4
Trustworthiness	3. The app mentions the references for the health information that it provides. Examples of trustworthy references are scientific papers or websites of the governmental health organizations, universities, or not-for-profit health groups.	0	1	2	3	4
Ease of use	4. Moving from one screen of the app to another would be easy for the user.	0	1	2	3	4
	5. It would be easy for the user to see components of the app such as text, icons, and buttons. **Pay attention to colours and sizes.**	0	1	2	3	4
	6. It would be easy for the user to understand the information provided by the app. **Pay attention to the text, graphs, tables, audio, or video.**	0	1	2	3	4
Functionality	7. The app components work correctly. For example, it does not crash or all links work.	0	1	2	3	4
	8. The user would be able to customize the app settings **(language, font size, font colour, background colour, reminders, and notifications)** to her/his satisfaction.	0	1	2	3	4
Target users	9. The content of the app is appropriate for the user considering her/his age, gender, education, and cultural background.	0	1	2	3	4
Usefulness and Satisfaction	10. The app can help the user to achieve her/his goals.	0	1	2	3	4
	11. The user would find the app pleasing to use.	0	1	2	3	4
	12. The user would be satisfied with using the app.	0	1	2	3	4
**Add up the scores for Part B: ……………………/48**						
Overall, I would recommend using this app to the user.		0	1	2	3	4
Circle the number of stars that best represents your overall rating for quality of this app: **(1 star =Worst app I have ever used; 5 Stars= Best app I have ever used)**		★	★	★	★	★

Alberta Rating Index for Apps (ARIA)- User version

App Name: ………………………………………….. I have used this app before: Yes ☐ No ☐

Use this index to rate the quality of a mobile health app. Please complete Part A and Part B.

**Part A:**<br>**Complete this part before downloading the app on your phone or tablet.**<br>• First, **find the app on your online app store**. For Apple products open "App store" for Android products go to the "Play Store."<br>• Then, read the app description from the online app store and rate the extent to which you agree with the following statements.		Strongly disagree	Disagree	Neutral	Agree	Strongly agree
Purpose	1. The description of the app's purpose fits my goals. **(You may find app description under "About this app" on Google Play; or "Preview" on iTunes)**	0	1	2	3	4
Trustworthiness	2. Based on the description provided on the app store or the app developer's website, I trust that relevant experts in the field have developed the app.	0	1	2	3	4
	3. The app description includes a statement about the risks associated with using the app.	0	1	2	3	4
	4. The app declares conflicts of interest, if any.	0	1	2	3	4
Privacy	5. The app has a privacy policy that explains: (1) what information is collected by the app, (2) who will have access to this information, and (3) how this information will be used. **(Look for the "Privacy policy" of the app under the app description on "App Store" for Apple products or "Play store" for Android products)**	0	1	2	3	4
Affordability	6. The costs associated with using the app, including in-app purchases and subscription renewal fees, are affordable. **(Go to the "App Store" for Apple products or "Play store" for Android products to learn how much does it cost to use the app)**	0	1	2	3	4
**Add up the scores for Part A**:……………………/24						

	**Part B:** Use the app for at least 10 minutes or as long as you feel it is necessary to become familiar with its features. Try all **the links** and **buttons** on the screen. After you become familiar with the app, rate the extent to which you agree with the following statements. **You may go back to the app and check the features**.	Strongly disagree	Disagree	Neutral	Agree	Strongly agree
Security	1. The app uses at least one security measure, such as **user name and password** or biometric identifiers **(fingerprints, face recognition)**, to allow users to access the app.	0	1	2	3	4
	2. The app asks for my consent if it needs to access the phone's camera, microphone, my location, my contacts, or my photos.	0	1	2	3	4
Trustworthiness	3. The app mentions the references for the health information that it provides. Examples of trustworthy references are scientific papers or websites of the governmental health organizations, universities, or not-for-profit health groups.	0	1	2	3	4
Ease of use	4. Moving from one screen of the app to another is easy for me.	0	1	2	3	4
	5. It is easy for me to see components of the app such as text, icons, and buttons. **Pay attention to colours and sizes.**	0	1	2	3	4
	6. It is easy for me to understand the information provided by the app. **Pay attention to the text, graphs, tables, audio, or video.**	0	1	2	3	4
Functionality	7. The app components work correctly. For example, it does not crash or all links work.	0	1	2	3	4
	8. I can customize the app settings to my satisfaction **(Try to customize language, font size, font colour, background colour, reminders, and notifications).**	0	1	2	3	4
Target users	9. The content of the app is appropriate for me considering my age, gender, education, and cultural background.	0	1	2	3	4
Usefulness and Satisfaction	10. The app can help me to achieve my goals.	0	1	2	3	4
	11. The app is pleasing to use.	0	1	2	3	4
	12. I am satisfied with using the app.	0	1	2	3	4
**Add up the scores for Part B**:……………………/48						
Circle the number of stars that best represents your overall rating for quality of this app: **(1 star =Worst app I have ever used; 5 Stars= Best app I have ever used)**		★	★	★	★	★

Indice d’évaluation de la qualité des applications de santé mobiles (version pour les fournisseurs de soins)

Nom de l’application : …………………………………………..   J’ai déjà utilisé cette application : Oui ☐ Non ☐

Utilisez ce tableau pour évaluer la qualité d’une application de santé mobile chez un client ou un patient (ci-après, « utilisateur »). Merci de remplir les Parties A et B.

Partie A: **Remplir cette partie avant de télécharger l’application sur votre téléphone ou votre tablette.** • Tout d’abord, **trouvez l’application en ligne**. Pour les produits Apple, ouvrez « App store »; pour les produits Android, rendez-vous au « Play Store ». • Puis, lisez la description de l’application et indiquez dans quelle mesure vous êtes d’accord (ou non) avec les énoncés suivants.		Fortement en désaccord	Pas d’ accord	Neutre	D’ accord	Fortement d’ accord
Objectif	1. La description de l’objectif de l’application convient aux objectifs de l’utilisateur. **(vous trouverez la description de l’application sous « About this app » *(Au sujet de cette app)* dans Google Play; ou sous « Preview » *(Aperçu)* dans iTunes)**	0	1	2	3	4
Fiabilité	2. Selon la description fournie dans l’App Store ou sur le site Web du développeur, l’utilisateur peut avoir confiance que l’application a été développée par des experts compétents dans le domaine.	0	1	2	3	4
	3. La description de l’application comprend un énoncé portant sur les risques associés à l’utilisation de l’application.	0	1	2	3	4
	4. Le cas échéant, les conflits d’intérêts sont énoncés.	0	1	2	3	4
Confidentialité	5. La politique de confidentialité de l’application énonce : (1) les renseignements recueillis par l’application; (2) qui aura accès à ces informations; et (3) comment ces informations seront utilisées. **(recherchez la politique de confidentialité dans la description de l’application dans l’App Store pour les produits Apple ou dans le Play store pour les produits Android)**	0	1	2	3	4
Abordabilité	6. Les coûts associés à l’utilisation de l’application, y compris les achats intégrés et les frais de renouvellement d’adhésion, sont abordables pour l’utilisateur. **(rendez-vous dans l’App Store pour les produits Apple ou dans le Play store pour les produits Android pour savoir combien coûte l’utilisation de l’application)**	0	1	2	3	4
**Additionnez les scores de la Partie A**:……………………/24						

Partie B : Utilisez l'application pendant au moins 10 minutes, ou pendant aussi longtemps que vous l'estimez nécessaire pour vous familiariser à ses caractéristiques. Essayez tous **les liens** et **les boutons** sur l'écran. Après vous être familiarisé avec l'application, évaluez la mesure dans laquelle vous êtes d'accord ou non avec les énoncés suivants. **Vous pouvez retourner à l'application et vérifier ses caractéristiques**.

		Fortement en désaccord	Pas d' accord	Neutre	D' accord	Fortement d' accord
Sécurité	1. L'application est munie d'au moins une mesure de sécurité, comme un **nom d'utilisateur et un mot de passe,** ou encore des identificateurs biométriques **(empreintes digitales, reconnaissance faciale)** qui permettent à l'utilisateur d'accéder à l'application.	0	1	2	3	4
	2. L'application demande le consentement de l'utilisateur si elle doit accéder à sa caméra, son micro, son emplacement, ses contacts ou ses photos.	0	1	2	3	4
Fiabilité	3. L'application indique les références des informations relatives à la santé qui y sont mentionnées. Les exemples de références fiables sont les articles scientifiques ou les sites Web des organismes de santé gouvernementaux, les universités ou les groupes sans but lucratif œuvrant dans le domaine de la santé.	0	1	2	3	4
Facilité d' utilisation	4. L'utilisateur passera facilement d'un écran à l'autre de l'application.	0	1	2	3	4
	5. L'utilisateur verra facilement les éléments de l'application, comme le texte, les icônes et les boutons. **Faites attention aux couleurs et aux tailles.**	0	1	2	3	4
	6. L'utilisateur comprendra facilement les informations fournies par l'application. **Faites attention au texte, aux graphiques, aux tableaux, à l'audio ou à la vidéo.**	0	1	2	3	4
Fonctionnalité	7. Les éléments de l'application fonctionnent correctement. Par exemple, le système ne plante pas et tous les liens fonctionnent correctement.	0	1	2	3	4
	8. L'utilisateur pourra personnaliser les paramètres de l'application **(langue, taille et couleur de la police, couleur de l'arrière-plan, rappels et notifications)** à son goût.	0	1	2	3	4
Utilisateur s ciblés	9. Le contenu de l'application est approprié pour l'utilisateur, compte tenu de son âge, son sexe, sa formation et de ses antécédents culturels.	0	1	2	3	4
Utilité et satisfaction	10. L'application peut aider l'utilisateur à atteindre ses objectifs.	0	1	2	3	4
	11. L'utilisateur trouvera l'application agréable à utiliser.	0	1	2	3	4
	12. L'utilisateur sera satisfait d'utiliser l'application.	0	1	2	3	4
**Additionnez les scores de la Partie B : ……………………/48**						
En général, je recommanderais cette application à l'utilisateur.		0	1	2	3	4
Entourez le nombre d'étoiles qui représente au mieux votre évaluation générale de la qualité de l'application : **(1 étoile = La pire application que j'ai jamais utilisée; 5 étoiles = La meilleure application que j'ai jamais utilisée)**		★	★	★	★	★

Indice d’évaluation de la qualité des applications de santé mobiles (version pour les utilisateurs)

Nom de l’application ………………………………………….. J’ai déjà utilisé cette application : Oui □ Non □

Utilisez ce tableau pour évaluer la qualité d’une application de santé mobile. Merci de remplir les Parties A et B.

	**Partie A:**<br>**Remplir cette partie avant de télécharger l’application sur votre téléphone ou votre tablette.**<br>• Tout d’abord, **trouvez l’application en ligne**. Pour les produits Apple, ouvrez « App store »; pour les produits Android, rendez-vous au « Play Store »<br>• Puis, lisez la description de l’application et indiquez dans quelle mesure vous êtes d’accord (ou non) avec les énoncés suivants.	Fortement en désaccord	Pas d’ accord	Neutre	D’ accord	Fortement d’ accord
Objectif	1. La description de l’objectif de l’application répond à mes objectifs. **(vous trouverez la description de l’application sous « About this app » (Au sujet de cette app) dans Google Play; ou sous « Preview » (Aperçu) dans iTunes)**	0	1	2	3	4
Fiabilité	2. Selon la description fournie dans l’App Store ou sur le site Web du développeur, j’ai confiance que l’application a été développée par des experts compétents dans le domaine.	0	1	2	3	4
	3. La description de l’application comprend un énoncé portant sur les risques associés à l’utilisation de l’application.	0	1	2	3	4
	4. Le cas échéant, les conflits d’intérêts sont énoncés.	0	1	2	3	4
Confidentialité	5. La politique de confidentialité de l’application énonce : (1) les renseignements recueillis par l’application; (2) qui aura accès à ces informations; et (3) comment ces informations seront utilisées. (**recherchez la politique de confidentialité dans la description de l’application dans l’App Store pour les produits Apple ou dans le Play store pour les produits Android)**	0	1	2	3	4
Abordabilité	6. Les coûts associés à l’utilisation de l’application, y compris les achats intégrés et les frais de renouvellement d’adhésion, sont abordables. **(rendez-vous dans l’App Store pour les produits Apple ou dans le Play store pour les produits Android pour savoir combien coûte l’utilisation de l’application)**	0	1	2	3	4
**Additionnez les scores de la Partie A**:……………………/24						

	**Partie B :** Utilisez l'application pendant au moins 10 minutes, ou pendant aussi longtemps que vous l'estimez nécessaire pour vous familiariser à ses caractéristiques. Essayez tous **les liens** et **les boutons** sur l'écran. Après vous être familiarisé avec l'application, évaluez la mesure dans laquelle vous êtes d'accord ou non avec les énoncés suivants. **Vous pouvez retourner à l'application et vérifier ses caractéristiques**.	Fortement en désaccord	Pas d' accord	Neutre	D' accord	Fortement d' accord
Sécurité	1. L'application est munie d'au moins une mesure de sécurité, comme **un nom d'utilisateur et un mot de passe**, ou encore des identificateurs biométriques **(empreintes digitales, reconnaissance faciale)** qui permettent aux utilisateurs d'accéder à l'application.	0	1	2	3	4
	2. L'application demande mon consentement si elle doit accéder à ma caméra, mon micro, mon emplacement, mes contacts ou mes photos.	0	1	2	3	4
Fiabilité	3. L'application indique les références des informations relatives à la santé qui y sont mentionnées. Les exemples de références fiables sont les articles scientifiques ou les sites Web des organismes de santé gouvernementaux, les universités ou les groupes sans but lucratif œuvrant dans le domaine de la santé.	0	1	2	3	4
Facilité d' utilisation	4. Je trouve qu'il est facile de passer d'un écran à l'autre de l'application.	0	1	2	3	4
	5. Je trouve qu'il est facile de voir les éléments de l'application, comme le texte, les icônes et les boutons. **Faites attention aux couleurs et aux tailles.**	0	1	2	3	4
	6. Je trouve qu'il est facile de comprendre les informations fournies par l'application. **Faites attention au texte, aux graphiques, aux tableaux, à l'audio ou à la vidéo.**	0	1	2	3	4
Fonctionnalité	7. Les éléments de l'application fonctionnent correctement. Par exemple, le système ne plante pas et tous les liens fonctionnent correctement.	0	1	2	3	4
	8. Je peux personnaliser les paramètres de l'application **(essayez de personnaliser la langue, la taille et la couleur de la police, la couleur de l'arrière-plan, les rappels et les notifications)** à mon goût**).**	0	1	2	3	4
Utilisateur s ciblés	9. Le contenu de l'application est approprié pour moi, compte tenu de mon âge, mon sexe, ma formation et de mes antécédents culturels.	0	1	2	3	4
Utilité et satisfaction	10. L'application peut m'aider à atteindre mes objectifs.	0	1	2	3	4
	11. L'application est agréable à utiliser.	0	1	2	3	4
	12. Je suis satisfait de l'utilisation de l'application.	0	1	2	3	4
	**Additionnez les scores de la Partie B**:……………………./48					
	Entourez le nombre d'étoiles qui représente au mieux votre évaluation générale de la qualité de l'application: **(1 étoile = La pire application que j'ai jamais utilisée; 5 étoiles = La meilleure application que j'ai jamais utilisée)**	★ ★ ★ ★ ★				
