# Supplementary material for: Efficacy, Effectiveness, and Quality of Resilience-Building Mobile Health Apps for Military, Veteran, and Public Safety Personnel Populations: Scoping Literature Review and App Evaluation
Source: JMIR Mhealth Uhealth. 2022 Jan 19;10(1):e26453. doi: 10.2196/26453 (PMC8811698; doi:10.2196/26453)
Supplement: Multimedia Appendix 3 [file mhealth_v10i1e26453_app3.pdf]

Multimedia Appendix 3: ARIA Score Table

	Part A					
		Trustworthiness				
	Purpose	Relevant Experts	Risks	Conflicts of Interest	Privacy	Affordability
AIMS for Anger Management	4	4	1	1	4	4
Breathe2Relax	4	2	1	1	1	3
CBT-i Coach	3	4	3	1	4	4
Daily Yoga	4	3	3	1	4	1
DoD Safe Helpline	3	3	2	2	4	4
eQuoo	3	4	1	1	4	3
High Res	3	3	1	1	0	4
Life Armor	4	1	1	1	1	4
MilTeenChat	4	3	2	1	4	4
Mindarma	2	3	1	1	1	3
Mindfulness Coach	4	3	1	1	4	3
PE Coach 2	3	3	2	1	4	4
PHIT for Duty	4	2	3	1	1	4
Positive Activity Jackpot	3	3	2	1	0	4
PTSD Coach	4	3	1	1	4	4
PTSD Family Coach	4	3	1	1	4	4

R2MR	4	3	1	1	4	4
Simply Yoga	3	2	3	1	4	2
Stay Quit Coach	3	4	0	1	4	4
T2 Mood Tracker	3	2	1	1	0	4
Tactical Breather	3	3	3	1	2	4
Virtual Hope Box	4	2	0	1	0	4

	Part B											
	Security			Ease of Use			Functionality			Usefulness and Satisfaction		
	Security Measures	User Consent	Trustworthiness	Moving between screens	Visual Clarity	Information Clarity	Components work correctly	Customization	Target Users	Can support goals	Pleasing to use	Satisfaction
AIMS for Anger Management	3	4	3	3	4	3	2	2	3	4	4	3
Breathe2Relax	1	1	2	3	3	3	1	2	3	3	2	2
CBT-i Coach	1	3	4	3	3	3	4	2	4	3	3	3
Daily Yoga	4	4	1	3	3	3	4	3	4	4	3	3
DoD Safe Helpline	3	1	3	4	3	3	2	1	3	4	3	3
eQuoo	0	2	4	3	3	3	4	2	2	3	3	3
High Res	1	1	2	2	3	2	3	2	3	3	3	2
Life Armor	4	4	3	2	2	3	3	1	3	3	2	2

MilTeenChat	3	4	2	3	3	3	2	1	3	3	3	3
Mindarma	1	1	3	3	2	3	2	1	3	2	3	2
Mindfulness Coach	1	3	3	3	4	3	3	2	3	4	4	3
PE Coach 2	2	3	3	4	3	2	4	2	3	3	3	3
PHIT for Duty	0	3	4	2	2	2	3	1	3	3	1	2
Positive Activity Jackpot	4	4	3	2	2	3	2	0	3	3	3	3
PTSD Coach	1	4	3	3	3	3	4	3	4	4	4	3
PTSD Family Coach	2	3	4	3	4	3	4	2	3	3	4	3
R2MR	1	4	4	3	3	3	4	4	4	4	3	3
Simply Yoga	1	4	2	3	2	3	3	3	3	3	2	2
Stay Quit Coach	1	2	4	3	3	2	4	0	3	2	2	2
T2 Mood Tracker	3	3	3	3	3	3	0	3	4	3	3	3
Tactical Breather	1	4	3	3	3	3	2	2	4	3	2	2
Virtual Hope Box	4	4	1	3	3	2	1	2	3	3	2	3
